# eHealth Applications Improve Glycemic Control in Patients With Diabetes: Randomized Controlled Trial

**DOI:** 10.2196/67761

**Published:** 2025-09-26

**Authors:** Junjie Huang, Claire Chenwen Zhong, Siu Hin Wong, Chung Yi Lo, Man Kin Yim, Martin C S Wong

**Affiliations:** 1 The Jockey Club School of Public Health and Primary Care Faculty of Medicine Chinese University of Hong Kong Hong Kong China (Hong Kong); 2 Centre for Health Education and Health Promotion Faculty of Medicine The Chinese University of Hong Kong Hong Kong China (Hong Kong); 3 The School of Public Health Peking University Beijing China; 4 The School of Public Health The Chinese Academy of Medical Sciences and The Peking Union Medical Colleges Beijing China; 5 The School of Public Health Fudan University Shanghai China

**Keywords:** randomized controlled trial, hemoglobin A1c, eHealth chronic disease management module, glycemic control, diabetes

## Abstract

**Background:**

The eHealth app introduced a chronic disease management module to enhance the efficiency of clinical consultations and facilitate self-health management. However, there was limited information on health outcomes after using the module.

**Objective:**

This study aimed to examine the health outcomes of individuals who used the health management module of an individualized electronic application compared to those who did not.

**Methods:**

A randomized controlled trial was conducted with 165 participants, with 82 participants assigned to the control group and 83 participants assigned to the intervention group. Randomization was done via a computer randomizer to evaluate the impact of the eHealth chronic disease management module installation on clinical outcomes such as blood pressure, hemoglobin A1c (HbA1c), renal function tests, estimated glomerular filtration rate, and urine albumin/creatinine ratio. Data were collected at baseline and at follow-up visits at 4 and 8 months. Student *t* tests and chi-square tests were performed to analyze the difference between the intervention and control groups and examined the potential impact of the use of the eHealth chronic disease management module on various health outcomes.

**Results:**

In total, 161 participants were included in the analysis, with an average age of 66.58 (SD 9.75) and 66.49 (SD 8.45) years in control and intervention group respectively. After 4 months, the intervention group showed better glycemic control, with significantly lower mean HbA1c levels (mean 6.76%, SD 0.64%) compared to the control group (mean 7.09%, SD 0.82%, *P*=.007). Also, more participants in intervention group achieved optimal HbA1c levels (n=58, 73.4%; *P=*.004) compared to the control group (n=36, 49.3%) in month 4. App usage had significantly decreased when comparing the usage after 4 months (mean 1.88 points, SD 0.81 points) and month 8 (mean 1.39 points, SD 0.72 points; *P*<.001). The results indicated better glycemic control for participants using the module in a relatively shorter period of time, and app adherence was the key for the continuous optimal glycemic control.

**Conclusions:**

These findings support the potential of the module for clinical application in patients with suboptimal glycemic control. The long-term benefit of the module may be affected by the compliance of participants to the module.

**Trial Registration:**

Chinese Clinical Trial Register ChiCTR2500108895; https://www.chictr.org.cn/showprojEN.html?proj=214865

## Introduction

The primary goal of eHealth is to leverage information and communication technology to improve health care outcomes [1]. This includes promoting healthy behaviors, supporting self-management of chronic diseases, and facilitating communication between patients and health care providers [2,3]. The Food and Drug Administration (FDA) defined a mobile medical app as an app used as an accessory to a regulated medical device [4]. The FDA released guidance to regulate mobile medical apps and ensured the safety of these apps for the user [5]. By using eHealth apps, patients can access crucial information, monitor their physical and mental well-being, and make informed health care decisions [6-10]. Some studies have demonstrated the positive impact of eHealth apps on specific conditions, such as diabetes self-management [11]. The use of mobile apps is associated with improved attitudes toward disease self-management, as well as significant improvements in hemoglobin A1c (HbA1c) levels, diabetes knowledge, and self-care behaviors [11,12].

In Hong Kong, the Food and Health Bureau and the Hospital Authority have developed an electronic health record sharing system (eHRSS) and an eHealth app. The eHRSS enables the sharing of electronic health records between public and private health care providers [13]. The number of beneficiaries from this computerized platform was numerous, including private hospitals and clinics, the Department of Health, primary care providers and clinicians, nursing colleagues, allied health professionals, and pathologists [14]. The health management module of eHealth was subsequently launched in July 2021 to enhance patient engagement and self-monitoring. This module enables users to record and monitor vital health indicators such as blood pressure, blood glycemic level, and heart rate, and provides reminders for regular measurements [15]. Additionally, patients can download and share their data or compile personal health reports for reference by health professionals and their family members. Hence, this may enhance the efficiency of clinical consultations and facilitate health management by patients and family members.

Given the growing role of eHealth in chronic disease management, this study seeks to determine whether the use of this module enhances patient engagement and self-monitoring, leading to better clinical outcomes compared to a control group without the app. Specifically, we conducted a randomized controlled trial to evaluate the effectiveness of the eHealth chronic disease management module in improving key health outcomes, including BMI, blood pressure, HbA1c, and urine albumin/creatinine ratio in patients with diabetes in a general outpatient clinic over a 1-year period.

## Methods

### Ethical Considerations

This study was approved by The Joint Chinese University of Hong Kong – New Territories East Cluster Clinical Research Ethics Committee (2022.264), Hong Kong SAR. All participants were informed of the voluntary and anonymous nature of the study. Written consent was obtained from all participants. Participants were given a document to explain the study’s purpose and procedures. All data collected were anonymized to ensure that there was no personally identifiable information, encrypted, and stored electronically.

### Study Design

This randomized controlled trial was conducted over a period of 1 year in 2024, involving 165 participants, with a follow-up time of 8 months. A total of 83 participants were assigned to the intervention group and 82 participants to the control group using a computer-generated randomization process. Patients were categorized into 2 groups: those who had adopted the health management module and those who had not. The intervention group received the eHealth app, which was installed on their phones with detailed instructions from our team members at baseline. Reminders were sent weekly to ensure the use of the app among participants in the intervention group. During the designated reporting period, 3 to 6 phone calls were conducted to remind participants for blood tests. The enrollment process adhered to the CONSORT (Consolidated Standards of Reporting Trials) guidelines [16].

### Participants

This trial was performed in a designated general outpatient clinic with a high volume of patients with diabetes attending for follow-up. Patients’ eHRSS registration status was documented in the clinical management system. Eligible participants included (1) adult patients aged 18 years or older, (2) patients with physician-diagnosed diabetes who had undergone diabetes mellitus complication screening before their installation of the eHRSS health management module, (3) regular visitors of the study clinic, and (4) patients diagnosed with type 2 diabetes. Exclusion criteria were the presence of medical conditions that impaired their ability to understand and participate in the study and previous use of the module.

### Procedures

Eligible participants were recruited in the clinic and underwent a subsequent diabetes mellitus complication screening to assess the potential complications of diabetes. Social demographic information was collected, including information on the patients’ lifestyles, such as dietary habits, physical activity, cigarette smoking, and alcohol consumption. Their clinical parameters were also assessed, including BMI, blood pressure levels, HbA1c levels, urinalysis (albumin/creatinine ratio), and the presence of diabetes complications.

### Outcomes

Data were collected at baseline and at follow-up visits at 4 and 8 months. Recruited participants underwent assessments for various diabetes-related health parameters, including blood pressure, HbA1c, renal function tests, and urine albumin/creatinine ratio. Additionally, lifestyle data including dietary consumption, physical activity, cigarette smoking, and alcohol consumption were collected. Dietary consumption and physical activity were measured using a 4-point Likert scale from 1 (never) to 4 (every time), with higher scores representing healthier lifestyles. Information on diabetes-related complications (macrovascular complications such as coronary artery disease, peripheral arterial disease, and stroke and microvascular complications such as diabetic neuropathic, nephropathy, and retinopathy) and prescribed medications was also collected. App usage at 4 and 8 months were measured using a 6-point Likert scale from 0 (never) to 5 (every time), with higher scores representing higher levels of use.

### Randomization

Block randomization was adopted in this study to ensure balanced group sizes throughout the trial using blocks of size 4. This approach allowed random assignment of participants within each block, helping to maintain equilibrium between treatment and control groups for potential confounding variables.

### Statistical Analysis

Baseline characteristics were compared between the intervention and control groups. Student *t* tests for continuous variables and chi-square tests for categorical variables were performed. Each clinical parameter was analyzed between the intervention and control group to examine the potential impact of the use of the eHealth chronic disease management module on various health outcomes. All analyses were performed using SPSS version 26.0 (IBM Corporation). *P* values <.05 were considered statistically significant.

## Results

### Characteristics of Participants

**Figure 1:**
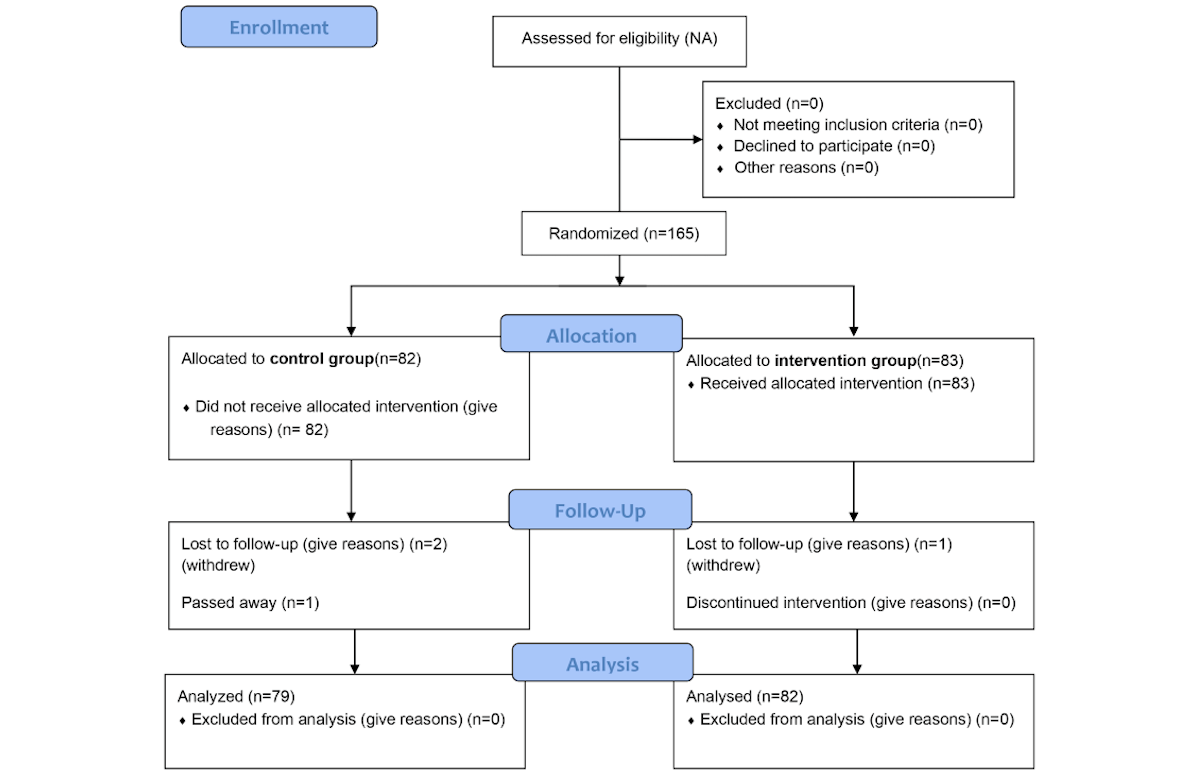
CONSORT (Consolidated Standards Of Reporting Trials) flow diagram.

A total of 165 patients were recruited, with 161 completing at least 1 follow-up (Figure 1). During the study, 4 participants were lost to follow-up, resulting in a default rate of 2.4%. In the end, 79 participants in control group and 82 participants in intervention group were included in the analysis. The average age of the participants was 66.58 (SD 9.75) years in the control group and 66.49 (SD 8.45) years in the intervention group, with no difference between groups. Details of patient sociodemographics are shown in Table 1.

At the 4-month follow-up visit, the intervention group exhibited better diabetes control, as indicated by a significant difference in HbA1c levels. The intervention group had a mean HbA1c of 6.76% (SD 0.64%), compared to 7.09% (SD 0.82%) in the control group (*P*=.007). However, for other outcome measures, such as renal function tests, estimated glomerular filtration rate, and urine albumin/creatinine ratio, no significant differences were observed between the 2 groups. Additionally, subsequent follow-ups did not reveal any significant differences (Table 2).

**Table 1:** Participant characteristics.

Demographics			Control		Intervention
Age (years), mean (SD)			66.58 (9.75)		66.49 (8.45)
**Sex, n (%)**					
	Male	37 (46)		36 (44)	
	Female	43 (54)		46 (56)	
**Education level, n (%)**					
	Basic	32 (45)		28 (40)	
	Middle	35 (49)		36 (51)	
	High	4 (6)		6 (9)	
**Employment status, n (%)**					
	Employed	21 (33)		26 (40)	
	Unemployed	32 (51)		35 (54)	
	Housekeeper	10 (16)		3 (5)	
	Others	0 (0)		1 (2)	
**Government subsidy, n (%)**					
	Yes	35 (49)		33 (48)	
	No	36 (51)		36 (52)	
**Monthly household income (HKD)^a^, n (%)**					
	<15,000	14 (74)		7 (50)	
	15,000-40,000	4 (21)		7 (50)	
	>40,000	1 (5)		0 (0)	
Dietary consumption^b^, mean (SD)			2.46 (0.55)		2.46 (0.55)
Physical activity^b^, mean (SD)			2.43 (1.15)		2.65 (1.03)
**Cigarette smoking status, n (%)**					
	Current smoker	7 (10)		5 (7)	
	Previous smoker	14 (20)		8 (12)	
	Never	49 (70)		56 (81)	
**Alcohol consumption, n (%)**					
	Current consumer	23 (33)		14 (20)	
	Previous consumer	4 (6)		4 (6)	
	Never	42 (61)		52 (74)	
**Chronic disease status, n (%)**					
	No chronic disease	1 (1)		6 (9)	
	One chronic disease	25 (35)		31 (45)	
	Multimorbidity	45 (63)		32 (46)	
BMI (kg/m^2^), mean (SD)			25.15 (4.33)		24.93 (4.88)
Height (cm), mean (SD)			161.67 (7.98)		160.24 (19.9)
Weight (kg), mean (SD)			65.61 (12.37)		66.32 (13.3)

^a^A currency exchange rate of HKD 1=US $7.80 is applicable.

^b^Measured on a 4-point Likert scale.

**Table 2:** Student’s t tests analysis on the continuous variables between control and intervention groups

Continuous variables	Control, mean (SD)	Intervention, mean (SD)	*P* value	*t* value	Cohen *d*
SBP^a^	130.17 (18.11)	128.13 (13.54)	.79	0.43	0.128
DBP^b^	76.03 (10.14)	74.76 (9.05)	.81	0.417	0.132
SBP (month 4)	129.81 (14.09)	127.46 (10.82)	.25	1.148	0.187
SBP (month 8)	128.11 (14.03)	127.96 (13.09)	.95	0.067	0.011
DBP (month 4)	72.89 (11.32)	73.65 (9.28)	.66	–0.448	–0.073
DBP (month 8)	71.42 (9.05)	72.45 (9.47)	.5	–0.682	–0.111
HbA1c^c^ (month 4)	7.09 (0.82)	6.76 (0.64)	.007	2.722	0.444
HbA1c (month 8)	6.83 (0.98)	6.89 (0.87)	.72	–0.365	–0.061
Sodium (mmol/L) (month 4)	139.54 (2.44)	139.46 (2.64)	.84	0.203	0.032
Sodium (mmol/L) (month 8)	139.71 (2.27)	138.96 (2.78)	.07	1.822	0.297
Potassium (mmol/L) (month 4)	4.20 (0.40)	4.29 (0.44)	.16	–1.401	–0.223
Potassium (mmol/L) (month 8)	4.17 (0.37)	4.31 (0.49)	.06	–1.904	–0.311
Urea (mmol/L) (month 4)	6.22 (2.06)	6.05 (1.64)	.56	0.578	0.088
Urea (mmol/L) (month 8)	6.65 (1.84)	6.14 (1.84)	.18	1.336	0.217
Creatinine (month 4) (µmol/L)	81.71 (26.97)	78.69 (22.97)	.45	0.761	0.121
Creatinine (month 8) (µmol/L)	86.75 (25.68)	82.87 (22.60)	.32	0.992	0.161
Urine albumin (month 4) (mg/d)	6.83 (15.99)	6.70 (20.29)	.97	0.037	0.007
Urine albumin (month 8) (mg/d)	8.10 (17.07)	7.07 (18.56)	.77	0.29	0.058

^a^SBP: systolic blood pressure (kPa).

^b^DBP: diastolic blood pressure (kPa).

^c^HbA1c: hemoglobin A1c (%).

In terms of the proportion of participants who achieved optimal glycemic control, the results indicated a significant difference in HbA1c levels at the 4-month follow-up between the intervention and control groups. Specifically, 75.95% (60/83) of the intervention group achieved an optimal result, compared to 58.75% (47/82) in the control group (*P*=.02). No significant differences were found for the other outcome parameters, such as estimated glomerular filtration rate and urine albumin/creatinine ratio, and no further differences were observed in the subsequent follow-up (Table 3).

App usage also declined over time, with the intervention group showing a mean usage of 1.88 points (SD 0.81 points) at the 4-month follow-up compared to 1.39 points (SD 0.72 points) at the 8-month follow-up (*P*<.001), suggesting a decrease in engagement over time (Table 4).

**Table 3:** Chi-square tests on the categorical variables between control and intervention groups.

Variable		Control group, n (%)	Intervention group, n (%)	Total, n (%)	*P* value		Effect size (Cramér V)	
**Sex (baseline)**						.76		0.069
	Female	43 (54)	46 (56)	89 (55)				
	Male	37 (46)	36 (44)	73 (45)				
**HbA1c^a^ (baseline)**						.86		0.016
	Optimal	41 (62)	43 (65)	84 (64)				
	Abnormal	25 (38)	23 (35)	48 (36)				
**HbA1c (month 4)**						0.004		0.234
	Optimal	36 (49)	58 (73)	94 (62)				
	Abnormal	37 (51)	21 (27)	58 (38)				
**HbA1c (month 8)**						.41		0.069
	Optimal	43 (58)	47 (66)	90 (62)				
	Abnormal	31 (42)	24 (34)	55 (38)				
**SBP^b^ (month 4)**						.15		0.118
	Optimal	54 (74)	67 (85)	121 (80)				
	Abnormal	19 (26)	12 (15)	31 (20)				
**DBP^c^ (month 4)**						>.99^d^		0
	Optimal	68 (93)	74 (94)	142 (93)				
	Abnormal	5 (7)	5 (6)	10 (7)				
**SBP (month 8)**						.66		0.036
	Optimal	59 (82)	67 (86)	126 (84)				
	Abnormal	13 (18)	11 (14)	24 (16)				
**DBP (month 8)**						>.99^d^		0
	Optimal	70 (97)	75 (96)	145 (97)				
	Abnormal	2 (3)	3 (4)	5 (3)				
**Creatinine^e^ (month 4)**						>.99		0
	Optimal	63 (81)	64 (82)	127 (81)				
	Abnormal	15 (19)	14 (18)	29 (19)				
**Creatinine (month 8)**						.51		0.054
	Optimal	58 (74)	57 (80)	115 (77)				
	Abnormal	20 (26)	14 (20)	34 (23)				
**eGFR^f^ (month 4)**						.34		0.08
	Optimal	54 (81)	65 (88)	119 (84)				
	Abnormal	13 (19)	9 (12)	22 (16)				
**eGFR (month 8)**						.37		0.077
	Optimal	48 (71)	52 (79)	100 (75)				
	Abnormal	20 (29)	14 (21)	34 (25)				
**Sodium^g^ (month 4)**						.52		0.051
	Optimal	76 (95)	72 (9)	148 (93)				
	Abnormal	4 (5)	7 (9)	11 (7)				
**Sodium (month 8)**						.25		0.094
	Optimal	77 (96)	65 (90)	142 (93)				
	Abnormal	3 (4)	7 (10)	10 (7)				
**Potassium^h^ (month 4)**						.97		0.003
	Optimal	75 (94)	72 (92)	147 (93)				
	Abnormal	5 (6)	6 (8)	11 (7)				
**Potassium (month 8)**						>.99		0
	Optimal	73 (91)	66 (92)	139 (91)				
	Abnormal	7 (9)	6 (8)	13 (9)				
**Urea^i^ (month 4)**						.76		0.024
	Optimal	57 (71)	59 (75)	116 (73)				
	Abnormal	23 (29)	20 (25)	43 (27)				
**Urea (month 8)**						.44		0.062
	Optimal	58 (73)	57 (79)	115 (76)				
	Abnormal	22 (28)	15 (21)	37 (24)				
**Urine albumin^j^ (month 4)**						.93		0.009
	Optimal	46 (94)	51 (96)	97 (95)				
	Abnormal	3 (6)	2 (4)	5 (5)				
**Urine albumin (month 8)**						.53		0.063
	Optimal	42 (91)	52 (96)	94 (94)				
	Abnormal	4 (9)	2 (4)	6 (6)				

^a^HbA1c: hemoglobin A1c; optimal level <7%.

^b^SBP: systolic blood pressure; optimal level <140 kPa.

^c^DBP: diastolic blood pressure; optimal level <90 kPa.

^d^Outcome of Fisher test.

^e^Optimal creatinine levels: 52.1-91.9 µmol/L for women and 65.4-119.3 µmol/L for men.

^f^eGFR: estimated glomerular filtration rate; optimal level ≥60 mL/min/1.73 m^2^.

^g^Optimal sodium level: 136-144 mmol/L.

^h^Optimal potassium level: 3.7-5.1 mmol/L.

^i^Optimal urea level: 2.1-7.1 mmol/L.

^j^Optimal urine albumin level: <30 mg/d.

**Table 4:** Student t test on app usage between month 4 and month 8 in the intervention group. App usage at 4 and 8 months was measured using a 6-point Likert scale from 0 (never) to 5 (every time), with a higher score representing a higher level of use.

Usage	Month 4_­_	Month 8	*P* value
Intervention usage, mean (SD)	1.88 (0.81)	1.39 (0.72)	<.001

## Discussion

### Brief Summary

This study found a significant improvement in HbA1c levels among participants in the intervention group at the 4-month follow-up and no significant difference between participants in the intervention group and control group at the 8-month follow-up. This finding suggests that optimal glycemic control could be achieved for short period of time with app use. However, the compliance of users with the module could be a barrier to the long-term success of app use on glycemic control and other health outcomes.

### Interpretations

This study showed short-term improvement of glycemic control, aligning with some previous studies. Tobacman et al [17] conducted a prospective cohort study and revealed a significant improvement in care service for the intervention group that had documentation of their own health records. Furthermore, better clinical outcomes were also observed from another randomized controlled trial study conducted by Bourgeois et al [18]. However, the phenomenon was not observed in month 8; this drop in the effect of the module could explain the decrease in medication adherence. Arshed et al [19] studied 23 studies on cardiovascular disease between 2000 and 2021 and revealed 26% of studies showed that mobile health interventions failed to show continuous impact on medication adherence. Pouls et al [20] conducted a systematic review and revealed that 17 out of the 29 randomized controlled trials included showed positive effects in medication adherence through use of SMS or interactive voice response, mobile apps, and phone calls as feedback, strategies to teaching, and facilitating communication or decision-making. The future of the management module could focus on developing features that facilitate increased adherence by patients, eventually improving clinical outcomes.

### Comparisons to Existing Literature

These findings were compatible with results from previous studies [21]. Many studies have pointed out that the use of electronic health programs can help patients improve their health behaviors and achieve better disease control, which may be attributed to the ability of the eHealth chronic disease management module to enhance self-control and self-efficacy, such as illness recognition and the stimulation of self-reflection through continuous monitoring of health data [22].

However, the lack of significant improvements in other clinical parameters and the diminished effect at the 8-month follow-up point to challenges in sustaining long-term engagement with the app. Our study also found that user engagement with the app declines over time. Previous studies also pointed out that users’ willingness to use the eHealth management module is often highest in the beginning stage, and the attrition rate may increase over time, potentially due to waning motivation, declining interest, and nonusage attrition [23,24]. This pattern may explain why the initial benefits observed at 4 months were not sustained at 8 months. Therefore, the sustainability of the apps’ long-term impact and continuous use need to be taken into consideration [25].

### Implications

Users of the eHealth app, particularly the eHealth management module, demonstrated better blood glucose control compared to nonusers. Older users showed better blood glucose control, despite the majority of app users being younger. Numerous factors, such as sociodemographic characteristics, medical conditions, duration of diabetes diagnosis, and medication, could have influenced the overall HbA1c outcomes. This study revealed no significant difference between the 2 groups in term of BMI, blood pressure, HbA1c, and urine albumin/creatinine ratio in patients with diabetes over a 1-year period. Developers of eHealth apps should focus on increasing the adherence of users to eHealth app usage and improving app features that may help patients to manage their weight, blood pressure, and chronic disease such as diabetes.

### Limitation

This study demonstrated the positive health outcomes in glycemic control achieved by using the eHealth chronic disease management module. However, this study has some limitations. First, despite reminders being sent routinely to participants in intervention group, participants may not have fully adhered to the modules, leading to biased results. Second, the statistically significant difference in HbA1c levels between the intervention and control groups may not represent a clinical difference. Further follow-up and investigation in real clinical settings are needed to confirm the finding. Third, based on the outcomes of this study, longer follow-up periods—such as 12 or 24 months—are suggested in order to generate a comprehensive result. Fourth, individuals assessed for eligibility were not included in the study, as recruitment occurred in the clinic without maintaining formal records of those assessed during the eligibility screening process.

### Conclusions

This randomized controlled trial demonstrated the potential short-term benefits of an eHealth chronic disease management module for improving diabetes control. However, the decline in app usage and lack of sustained improvement in other clinical parameters at the 8-month follow-up highlight the need for strategies to maintain user engagement over time. This study identified positive clinical outcomes from the module and the need for improvement in the compliance of patients. Future iterations of the eHealth program should incorporate features that enhance long-term user motivation, such as regular feedback, ongoing support, and educational interventions, particularly targeting older adults or those with limited technology proficiency. Ongoing evaluation and adaptation of the eHealth module will be critical to maximize its long-term impact on diabetes management.

## Abbreviations

CONSORT: Consolidated Standards of Reporting Trials
eHRSS: electronic health record sharing system
FDA: Food and Drug Administration
HbA1c: hemoglobin A1c

## Data Availability

The datasets used or analyzed during the current study are available from the corresponding author on reasonable request.
